# Cost-Effectiveness of Carbon Ion Radiotherapy in Oncology: A Systematic Review

**DOI:** 10.7759/cureus.84008

**Published:** 2025-05-13

**Authors:** Abdulrahman Bin Sumaida, Nandan M Shanbhag, Khalifa AlKaabi, Khalid Balaraj

**Affiliations:** 1 Oncology/Radiation Oncology, Tawam Hospital, Al Ain, ARE; 2 Radiation Oncology/Palliative Care, Tawam Hospital, Al Ain, ARE; 3 Internal Medicine, College of Medicine and Health Sciences, United Arab Emirates University, Al Ain, ARE; 4 Radiation Oncology, Tawam Hospital, Al Ain, ARE

**Keywords:** carbon ion radiotherapy, cost-effectiveness, health economics, oncology, particle therapy, recurrent cancer, systematic review

## Abstract

Carbon ion radiotherapy (CIRT) offers significant physical and biological advantages compared to conventional photon or proton therapies, particularly in the treatment of chemoradio-resistant tumors. However, the widespread adoption of CIRT has been limited due to high treatment and infrastructure costs. This systematic review evaluated the cost-effectiveness of CIRT relative to other treatment modalities across different cancer types. A structured literature search identified five eligible studies encompassing 486 patients with skull base chordoma, locally recurrent rectal cancer, localized hepatocellular carcinoma, and stage I non-small-cell lung cancer. Although CIRT was associated with higher primary treatment costs compared to conventional therapies, it consistently demonstrated favorable cost-effectiveness ratios, with incremental cost-effectiveness ranging from up to $88,663 per additional life-year or quality-adjusted life-year gained. In cases of recurrent tumors, CIRT resulted in lower overall treatment costs than comparator therapies. Additionally, CIRT exhibited a favorable toxicity profile, with minimal severe adverse events reported. These findings suggest that despite higher initial expenditures, CIRT represents a cost-effective and clinically advantageous treatment option across a range of malignancies, warranting broader consideration in oncologic practice.

## Introduction and background

Cancer remains a major global health burden, with an estimated 19.3 million new cases reported worldwide in 2020. This number is projected to rise by 47%, reaching approximately 28.4 million cases by 2040, largely driven by lifestyle and demographic shifts [1]. Among older adults, the burden is expected to increase even more sharply, with new cases projected to grow from 11.3 million in 2020 to 19.8 million by 2040 - a 75.2% increase [2]. The World Health Organization further estimates that by 2050, annual cancer incidence could surpass 35 million cases, with more than 18 million deaths disproportionately affecting low- and middle-income countries [3]. This alarming trend underscores the urgent need for therapies that are not only effective but also sustainable and accessible.

Carbon ion radiotherapy (CIRT) has emerged as a promising modality, particularly for tumors resistant to conventional chemoradiotherapy [4,5]. Utilizing synchrotron- or cyclotron-accelerated carbon ions, CIRT enables precise tumor targeting with minimal damage to surrounding normal tissues. The physical properties of carbon ions, particularly the pronounced Bragg peak, allow for superior dose conformity compared to photon or proton therapies [4,6,7]. Additionally, their high linear energy transfer (LET) results in greater relative biological effectiveness (RBE), producing complex and irreparable DNA damage even in hypoxic or radioresistant tumors [7,8]. Although carbon ions generate secondary fragments that slightly broaden the distal dose, the therapeutic ratio remains highly favorable.

Since its clinical introduction at the National Institute of Radiological Sciences (NIRS) in Japan in 1994, CIRT has been explored for a wide spectrum of malignancies, including intracranial tumors, head and neck cancers, lung cancers, gastrointestinal tumors, genitourinary cancers, soft tissue sarcomas, and recurrent disease [6]. By December 2020, approximately 37,500 patients had been treated at 12 CIRT centers across Asia and Europe [9]. Currently, around 13 active CIRT centers operate worldwide, mainly in Japan, Germany, China, Italy, and Austria [9-11]. Despite mounting clinical evidence demonstrating improved local control, progression-free survival, and overall survival compared to photon and proton therapies [12], CIRT remains underutilized globally. Meta-analyses have further confirmed that CIRT is associated with reduced rates of acute and late toxicities, including gastrointestinal and genitourinary side effects, while maintaining high tumor control rates [13,14]. Results from phase I/II studies, such as PROMETHEUS-01 for hepatocellular carcinoma (HCC) and PHOENIX-01 for pancreatic cancer, also highlight its efficacy and safety in challenging clinical scenarios [6].

Despite its robust clinical advantages, the expansion of CIRT has been hampered by significant economic barriers. In contrast to the approximately 80 operational proton therapy centers worldwide, with over 100 more under development [9,15,16], the number of CIRT centers remains limited to just over a dozen, with none currently established in the United States [9]. Financial considerations present the most formidable obstacle: constructing a comprehensive particle therapy facility that includes carbon ions costs upwards of €138.6 million, compared to €94.9 million for a proton-only center and €23.4 million for a photon facility [17]. Moreover, operational costs for CIRT remain substantially higher, with treatment costs reaching €1,128 per fraction, compared to €743 for protons and €233 for photons [17]. Additional indirect costs, including staffing, beam maintenance, and patient transportation, further escalate the financial burden.

This stark contrast between the clinical promise of CIRT and its economic demands presents a critical challenge for healthcare systems and policymakers. Stakeholders must balance the potential for improved survival and reduced toxicity against the high capital and operational costs associated with this technology. In light of these considerations, a comprehensive assessment of the cost-effectiveness of CIRT is essential. The objective of this systematic review is to synthesize available evidence on the economic evaluations of CIRT across various malignancies and to compare its value against established radiotherapy modalities.

## Review

Methods

This systematic review was conducted in accordance with the Preferred Reporting Items for Systematic Reviews and Meta-Analyses (PRISMA) 2020 guidelines [18]. The objective was to identify and synthesize published economic evaluations of CIRT compared to conventional or alternative radiotherapy modalities across various cancer types.

Information Sources and Search Strategy

A comprehensive literature search was performed across PubMed, Cochrane Central Register of Controlled Trials (CENTRAL), and Google Scholar from inception through April 1, 2025. Search terms included combinations of “carbon ion radiotherapy”, “CIRT”, “carbon ion therapy”, “particle therapy”, “cost-effectiveness”, “cost utility”, “economic evaluation”, “treatment cost”, and “health economics”. Boolean operators “AND” and “OR” were used to construct search strings. Full search strategies are provided in Table 1.

**Table 1:** Search strategy used in the systematic review CIRT: carbon ion radiotherapy

Database	Search String
PubMed/CENTRAL	(“carbon ion radiotherapy” OR “carbon ion therapy” OR CIRT OR “particle therapy”) AND (cancer OR “radioresistant cancer” OR “oncology” OR “tumor”) AND (cost OR “cost-effectiveness” OR “cost effectiveness” OR “cost utility” OR “economic evaluation” OR “health economics” OR “treatment cost” OR “recurrent cost”)
Google Scholar	(‘carbon ion radiotherapy’ OR ‘carbon ion therapy’ OR CIRT OR ‘particle therapy’) AND (cancer OR ‘radioresistant cancer’ OR ‘oncology’ OR ‘tumor’) AND (cost OR ‘cost-effectiveness’ OR ‘cost effectiveness’ OR ‘cost utility’ OR ‘economic evaluation’ OR ‘health economics’ OR ‘treatment cost’ OR ‘recurrent cost’)

Eligibility Criteria

Eligible studies were original research articles that performed a cost analysis of CIRT in cancer patients. Inclusion criteria were defined using the PICO framework: Population: patients with any type of cancer; Intervention: CIRT; Comparator: other standard cancer treatments, including photon radiotherapy, stereotactic body radiotherapy (SBRT), and multimodality therapies; Outcomes: direct or incremental treatment costs, cost-effectiveness ratio (CER), or incremental cost-effectiveness ratio (ICER).

Exclusion criteria included systematic reviews, meta-analyses, conference abstracts, modeling studies without patient-level data, editorials, author commentaries, and conceptual or hypothetical studies.

Study Selection and Data Extraction

All retrieved articles were imported into reference management software, and duplicates were removed. Title and abstract screening was followed by full-text review to assess eligibility. Two independent reviewers conducted the screening process, and discrepancies were resolved by consensus.

Data from included studies were extracted into a standardized table capturing study characteristics (e.g., country, year, population, cancer type), comparator treatment, total costs, ICERs, CERs, and toxicity outcomes. Where applicable, additional cost components such as direct versus indirect expenses and recurrent treatment costs were recorded.

Data Analysis

A structured narrative synthesis approach was used to summarize findings. Cost values were converted to 2024 US dollars using the CCEMG-EPPI-Centre Cost Converter v1.4 (Campbell and Cochrane Economics Methods Group and the Evidence for Policy and Practice Information and Co-ordinating Centre (CCEMG-EPPI-Centre), University College London, UK) [19], adjusted for inflation and purchasing power parity. Original currency values were retained in tables for reference. No meta-analysis was conducted due to heterogeneity in study designs, populations, and outcome reporting.

Results

Study Selection

The initial database search identified 621 articles: 515 from PubMed, six from CENTRAL, and 100 from Google Scholar. After removing 58 duplicates, 563 unique records were screened by title and abstract. Of these, 552 were excluded for topic irrelevance, as they primarily reported clinical outcomes without economic evaluation. Eleven full-text articles were assessed for eligibility, and six were excluded due to irrelevant outcomes (n=5) or being model-based studies without patient-level data (n=1). Ultimately, five studies met the inclusion criteria and were included in this review. All five studies included in this review employed a cost-effectiveness analysis (CEA) design. As all studies shared the same design, a separate column for study design was not included in the summary table to avoid redundancy.

**Figure 1:**
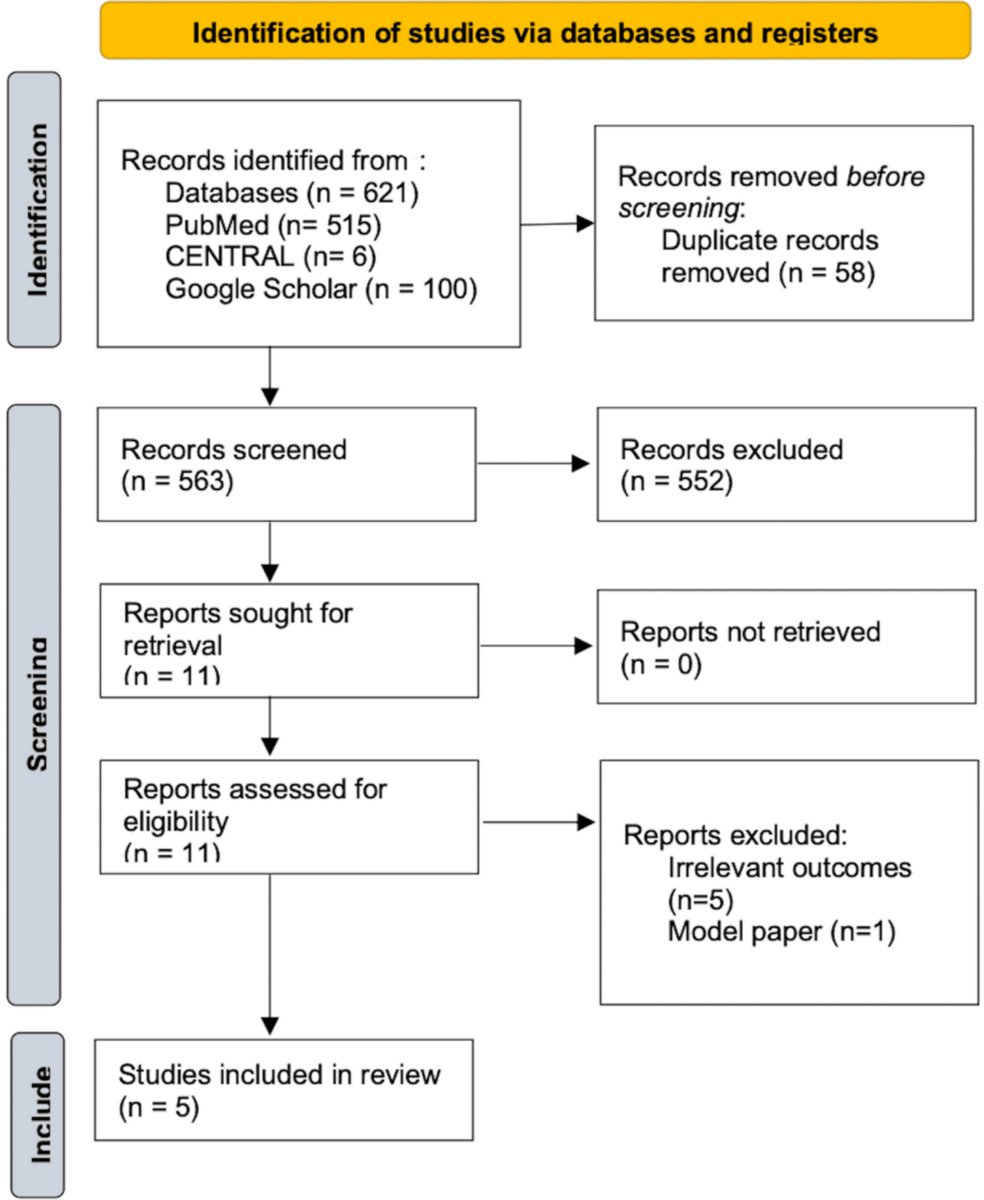
PRISMA flowchart This PRISMA flowchart summarizes the process of identifying, screening, assessing eligibility, and including studies for the systematic review. A total of 621 records were identified through database searches. After removing duplicates and screening titles, abstracts, and full texts, five studies met the inclusion criteria and were included in the final review. PRISMA: Preferred Reporting Items for Systematic Reviews and Meta-Analyses

The study selection process is illustrated in the PRISMA flowchart (Figure 1).

Study Characteristics

The five included studies were published between 2007 and 2024 and conducted in Germany and Japan [20-24]. Together, they analyzed a total of 486 patients across four cancer types: skull base chordoma, locally recurrent rectal cancer, localized HCC, and stage I non-small-cell lung cancer (NSCLC). Comparators included conventional photon radiotherapy, SBRT, transarterial chemoembolization (TACE), and multimodality treatments comprising radiotherapy, chemotherapy, and hyperthermia. Detailed characteristics of the included studies are summarized in Table 2.

**Table 2:** Characteristics of included studies evaluating the cost of carbon ion radiotherapy (CIRT) This table presents the study characteristics of the five included publications that evaluated the costs or cost-effectiveness of CIRT for various cancer types. Data includes the author(s), year of publication, country, cancer site, comparator treatment, number of patients, perspective of cost analysis, and primary outcome measures such as direct costs and ICERs. n: number of patients

Study	Country	Population	Cancer	Intervention vs. comparator treatment	Cost outcomes
Jäkel et al. (2007) [20]	Germany	10 patients	Skull base chordoma	CIRT vs. conventional radiotherapy (RT)	Treatment cost, cost-effectiveness ratio (CER)
Mobaraki et al. (2010) [21]	Japan	25 patients	Locally recurrent rectal cancer	CIRT (n=14) vs. conventional multimodality (three‐dimensional conformal radiotherapy, chemotherapy, and hyperthermia) therapy (n=11)	Incremental cost‐effectiveness ratio (ICER)
Okazaki et al. (2024) [22]	Japan	34 patients	Localized hepatocellular carcinoma	CIRT (n=17) vs. transarterial chemoembolization (TACE) (n=17)	Treatment cost
Okazaki et al. (2021) [23]	Japan	89 patients	Stage I non-small-cell lung cancer (NSCLC)	CIRT (n=62) vs. stereotactic body radiotherapy (SBRT) (n=27)	ICER
Sprave et al. (2018) [24]	Germany	328 patients	Skull base chordoma	CIRT vs. photon radiotherapy (PRT)	ICER

Primary Treatment Costs

Four of the five studies reported significantly higher primary treatment costs for CIRT compared to conventional therapies [20-22,24]. For example, Jäkel et al. [20] reported a total cost of USD 81,617 for CIRT versus USD 50,730 for conventional radiotherapy in skull base chordoma. Similarly, Okazaki et al. [22] observed that CIRT for stage I NSCLC cost USD 66,253 compared to USD 20,325 for SBRT. In contrast, Okazaki et al. [23] reported a slightly lower cost for CIRT (USD 54,970) than for TACE (USD 58,398) in treating localized HCC. Across studies, carbon ion beam generation represented a major portion of the treatment cost-over 65% in some cases [22,23].

Costs for Recurrent Tumors

For recurrent tumors, CIRT was generally less expensive than comparator therapies. Jäkel et al. [20] found that recurrent skull base chordoma treatment costs were lower for CIRT (USD 152,507) compared to conventional radiotherapy (USD 177,217). Similarly, in locally recurrent rectal cancer, Mobaraki et al. [21] reported additional treatment costs of USD 11,391 for CIRT versus USD 20,747 for multimodality therapy. Sprave et al. [24] found CIRT costs for recurrent skull base chordoma to be USD 213,516 versus USD 235,741 for photon radiotherapy.

Cost-Effectiveness

Four studies reported CERs or ICERs, demonstrating favorable economic outcomes for CIRT across cancer types [20-22,24]. For skull base chordoma, Jäkel et al. [20] estimated a CER of USD 4,753 per 1% increase in survival and USD 14,399 per additional life-year gained. In rectal cancer, Mobaraki et al. [21] reported a CER of USD 54 per life-year and USD 78 per 1% increase in survival. For stage I NSCLC, Okazaki et al. [22] reported an ICER of USD 88,663 per life-year. Sprave et al. [24] calculated a cost of USD 14,076 per quality-adjusted life-year (QALY), the only study to report a QALY-based CER. Table 3 presents the full summary of cost and effectiveness outcomes.

**Table 3:** Summary of cost-effectiveness outcomes for carbon ion radiotherapy (CIRT) This table summarizes the cost-effectiveness findings reported in the included studies on CIRT. Reported outcomes include the cost-effectiveness ratio (CER), life years (LY) gained, and quality-adjusted life years (QALYs), where available. All cost values are presented in United States Dollars (USD), with original costs in native currencies shown in brackets. USD values have been converted and rounded to the nearest dollar. “NR” indicates that the data was not reported by the study. RT: radiotherapy; TACE: transarterial chemoembolization; SBRT: stereotactic body radiotherapy

Study	Cancer	Total primary treatment cost	Total recurrent tumor cost	CER per 1% increase in survival	CER per additional life year	CER per additional year of disease-free survival
Jäkel et al. (2007) [20]	Skull base chordoma	81,617 USD vs. 50,730 USD (€ 43,600 vs. € 27,100) for conventional RT	152,507 USD vs. 177,217 USD (€ 81,470 vs. € 94,670)	4,753 USD (€ 2,539) per 1% increase in survival	14,399 USD (€ 7,692) per additional life year (33 years)	30,887 USD (€ 16,500) per year of disease-free survival
Mobaraki et al. (2010) [21]	Locally recurrent rectal cancer	58,418 USD vs. 56,073 USD (¥4,803,946 vs. ¥4,611,100) for the multimodality treatment group	11,391 USD vs. 20,747 USD (¥936,770 vs. ¥1,706,107) - additional cost	78 USD (¥6,428) per 1% increase in survival.	54 USD (¥4397) per additional life year (20 years)	164 USD (¥13,454) per year of disease‐free survival
Okazaki et al. (2024) [22]	Localized Hepatocellular Carcinoma	54,970 USD vs. 58,398 USD (¥4,974,278 vs. ¥5,284,524) for TACE	NR	NR	NR	NR
Okazaki et al. (2021) [23]	Stage I non-small-cell lung cancer (NSCLC)	66,253 USD vs. 20,325 USD (¥5,597,585 vs. ¥ 1,717,238) for SBRT	NR	NR	88,663 USD (¥ 7,491,017) per LY	NR
Sprave et al. (2018) [24]	Skull base chordoma	50,128 USD (€ 31,538.21)	213,516 USD vs. 235,741 USD (€ 134,335.97 vs. € 148,319.07)	NR	14,076 USD (€8,855.76) per QALY	NR

Toxicity

Only one study provided a detailed account of toxicity outcomes. In the study by Mobaraki et al., no patients in the CIRT group experienced grade ≥3 toxicity, with 93% experiencing only grade 1 skin reactions. In contrast, 27% of patients receiving multimodality therapy experienced grade 3 gastrointestinal toxicity. No urinary or hematologic toxicities were observed in either group [21].

Discussion

This systematic review confirms that CIRT, while associated with higher primary treatment costs than conventional modalities, demonstrates consistently favorable CERs across a range of malignancies, including skull base chordoma, locally recurrent rectal cancer, localized HCC, and stage I NSCLC. Notably, in the context of recurrent disease, CIRT often emerges as the less expensive option. Moreover, toxicity profiles across studies consistently favor CIRT, with minimal high-grade adverse events reported.

Jäkel et al. [20] reported primary treatment costs of USD 81,617 for CIRT compared to USD 50,730 for conventional radiotherapy in patients with skull-base chordoma. Similarly, Sprave et al. [24] found CIRT to incur direct costs of USD 30,132 - more than four times higher than those associated with photon radiotherapy (USD 7,470). In the treatment of stage I NSCLC, Okazaki et al. [22] observed that CIRT costs were approximately three times higher than those of SBRT (USD 66,253 vs. USD 20,325). These elevated costs mirror findings from broader economic analyses highlighting that CIRT is typically more expensive than conventional radiotherapy approaches [9,17]. However, in a propensity-matched study comparing CIRT and TACE for localized HCC, Okazaki et al. [23] reported that CIRT was actually less costly overall, illustrating how disease type and treatment context can significantly influence cost differentials.

While most studies agree that CIRT incurs higher costs, the degree of cost elevation varies depending on tumor type and clinical scenario. For example, Okazaki et al. [22] reported a threefold cost difference for stage I NSCLC, whereas Jäkel et al. [20] and Sprave et al. [24] reported more moderate differences in skull base chordomas.

In recurrent tumor settings, CIRT has been reported to be more economical than comparator treatments. This may be attributed to its superior local control rates and reduced need for retreatment or supportive care. For instance, Jäkel et al. [20] reported a two-year local control rate of 79% with CIRT versus 53% with conventional radiotherapy in recurrent skull base chordoma. Corresponding total costs were USD 152,507 for CIRT and USD 177,217 for conventional radiotherapy. The dosimetric advantages of CIRT, specifically the sharp dose fall-off and high LET, enable better sparing of normal tissues, which reduces toxicity and associated healthcare expenditures. In re-irradiation settings, retrospective studies have shown lower rates of acute and late ≥grade III toxicities with CIRT (3.1% and 14.5%, respectively) compared to photon-based techniques [6,24]. These reductions in treatment-related morbidity translate to fewer hospitalizations, reduced need for medications and supportive interventions, and shorter follow-up durations - all of which contribute to overall cost savings [6,15,24].

Despite its higher upfront costs, CIRT demonstrates favorable cost-effectiveness outcomes across various settings. For instance, Mobaraki et al. [21] reported an ICER of USD 78 per 1% increase in survival and USD 54 per life-year gained for recurrent rectal cancer. Similarly, Jäkel et al. [20] reported a CER of USD 4,753 per 1% survival gain and USD 14,399 per life-year for skull base chordoma. Sprave et al. [24] estimated a cost of USD 14,076 per QALY, while Okazaki et al. [23] demonstrated cost dominance of CIRT over TACE in localized HCC, with improved life-years (2.75 vs. 2.41) at a lower total cost (USD 54,970 vs. USD 58,398).

Toxicity data further support the cost-effectiveness of CIRT. In the present review, no patients experienced grade ≥3 late, urinary, or hematologic toxicity, and 93% reported only grade 1 skin reactions. These findings are consistent with meta-analytic data demonstrating significantly fewer grade 3+ adverse events with CIRT compared to photon or proton therapies (relative risk (RR)=0.77; 95% confidence interval (CI) 0.48-1.24) and fewer severe toxicities compared to non-particle therapies (RR=0.37; 95% CI 0.14-0.98) [7]. In addition to improved toxicity profiles, meta-analyses have also shown superior oncologic outcomes with CIRT, including improved 5-year overall survival (RR=1.19; 95% CI 1.01-1.42) and progression-free survival (RR=1.50; 95% CI 1.01-2.21) [7].

Limitations

This systematic review has limitations that should be acknowledged. First, the number of published studies evaluating the cost-effectiveness of CIRT remains limited, with only five studies meeting inclusion criteria. This restricts the generalizability of findings across diverse healthcare settings and cancer types. Second, all included studies were conducted in high-income countries, primarily Japan and Germany, which limits the applicability of economic outcomes to low- and middle-income settings where healthcare infrastructure and cost structures may differ significantly. Third, heterogeneity in study design, cancer types, costing perspectives (payer vs. societal), and outcome measures - such as life-years, QALYs, and ICERs - precluded a formal meta-analysis. Variations in exchange rates, time horizons, and discounting methods further complicate direct comparisons. Although currency conversions were standardized using a validated tool, cost estimates may still be influenced by differences in healthcare reimbursement models and inflation adjustments. Fourth, several studies lacked detailed reporting on indirect costs, long-term follow-up, or toxicity-related expenditures, which could lead to underestimation or overestimation of the true economic impact of CIRT. Additionally, the absence of randomized controlled trials (RCTs) in the included evidence base limits the strength of causal inferences regarding both clinical and economic superiority. Finally, publication bias cannot be excluded, as studies demonstrating favorable cost-effectiveness may be more likely to be published, while those showing neutral or unfavorable results may remain unpublished.

Implications for Future Research

This review highlights the scarcity of economic evaluations on CIRT, despite its clinical promise and rising global interest. The limited number of published cost-effectiveness studies, all from high-income countries, demands a pressing need for broader and more inclusive research. Future studies should focus on standardized economic methodologies, incorporate real-world data, and extend analyses to varied health system contexts, particularly in low- and middle-income countries. Additionally, integration of toxicity-related costs, patient-reported outcomes, and long-term follow-up will provide a more accurate assessment of CIRT’s value. As CIRT continues to expand, embedding economic endpoints within clinical trials will be essential to inform policy, justify investment, and optimize clinical use.

## Conclusions

This systematic review highlights the inherent tension between the substantial upfront capital and treatment costs of CIRT and its consistently favorable cost-effectiveness and safety profiles across multiple cancer types. For malignancies such as skull-base chordoma, recurrent rectal cancer, stage I NSCLC, and HCC, CIRT remains more expensive than conventional radiotherapy, SBRT, or multimodality approaches-primarily due to the high costs associated with carbon ion beam generation and facility infrastructure.

However, all included economic evaluations demonstrate that CIRT offers greater value in terms of survival gains, life-years, and QALYs. Its superior local control rates, capacity for hypofractionation, and significantly reduced toxicity- and retreatment-associated expenditures may, in select clinical contexts, offset initial costs and lead to net cost savings. These findings support the growing recognition of CIRT as a high-value therapeutic option, particularly in challenging or recurrent cancer settings where conventional modalities fall short.
